# Assessing metabolic rates in zebrafish using a 3D-printed intermittent-flow respirometer and swim tunnel system

**DOI:** 10.1242/bio.060375

**Published:** 2024-06-18

**Authors:** Rasmus Hejlesen, Freja Burkarl Scheffler, Clara Garfiel Byrge, Kasper Kjær-Sørensen, Claus Oxvig, Angela Fago, Hans Malte

**Affiliations:** ^1^ Department of Biology, Zoophysiology, Aarhus University, Aarhus DK-08000, Denmark; ^2^ Department of Molecular Biology and Genetics, Aarhus University, Aarhus DK-08000, Denmark

**Keywords:** Respirometer, Respirometry, Standard metabolic rate, Maximal metabolic rate, Aerobic scope, Fish

## Abstract

Zebrafish have become a widely used vertebrate model in physiology and reliable measures of their metabolic rate are needed. We have developed a 3D-printed respirometer and swim tunnel system and used it for obtaining accurate measurement of standard metabolic rate (SMR) and maximal, aerobic metabolic rate (MMR) in zebrafish under rest and maximal exercise, respectively. We compared a slow (stepwise) protocol to a fast (continuous) protocol for determining MMR. The fast protocol yielded slightly (but not significantly) higher oxygen consumption rates than the slow protocol and the data, in contrast to the slow protocol, followed a normal distribution. These findings point to the fast protocol as a fast and reliable method for obtaining accurate values of MMR in zebrafish. We make the 3D drawings for printing the system available to researchers, to help streamline the field of metabolic research in zebrafish and other smaller fish species.

## Teaser

**Summary:** Using a 3D-printed respirometer and swim tunnel system (drawings are available) we measured standard metabolic rate and compared two different protocols for measuring maximum, aerobic metabolic rate of zebrafish.

## INTRODUCTION

The metabolic rate of any animal reflects its cost of living. For endotherms the minimal metabolic rate (basal metabolic rate, BMR) is the resting metabolic rate of the fasting animal in its thermoneutral zone. For ectotherms, a resting and fasting animal is at its standard metabolic rate (SMR), which is temperature dependent. In either case, the BMR or SMR set the minimum food and energy requirement for survival and are useful physiological parameters for comparison of metabolic rates across species under identical conditions.

The maximal aerobic metabolic rate (MMR), also called aerobic capacity, is an important performance parameter used in the study of aerobic activity and reflects the maximal rate of ATP production by aerobic metabolism. A high MMR allows for a high sustained speed during persistent physical performance, which may allow to catch prey and/or avoid predators. In humans, MMR also serves as an important indicator of physical health. Because of the increased maintenance cost of a large metabolic engine, the evolution of a high MMR may have been the key driver for the evolution of a high SMR and BMR and ultimately for endothermy (Bennett and Ruben, 1979).

The difference between MMR and SMR is the absolute aerobic scope (AAS), which is the aerobic metabolic rate an ectotherm can spend on life processes like reproduction, growth and movement. It is widely considered to be the ultimate measure of aerobic performance (Fry and Hart, 1948) and shows large variation between individuals (Norin and Malte, 2011, 2012; Boldsen et al., 2013). While the difference between MMR and SMR describes the aerobic scope of the individual, the ratio between MMR and SMR, the so-called factorial aerobic scope, is often relevant in comparative studies and tells how many fold an organism can increase its aerobic metabolic rate, irrespective of the SMR.

The MMR is normally measured in fishes in one of two ways. One approach is measuring the O_2_ consumption rate immediately after exhaustion of the fish in a post-exhaustion experiment. This is done by chasing or by swimming in a flume (Norin and Clark, 2016), followed by rapid transfer of the fish to a respirometer and immediate start of O_2_ consumption measurements.

Another approach involves continuously monitoring O_2_ consumption in a respirometer while progressively increasing swimming speed until exhaustion. This method traditionally used a step-by-step protocol and its goal was to determine the maximal sustainable swimming speed that can be kept for a prolonged (typically 30 min) period of time (Brett, 1964; 1967; Beamish, 1978). This speed is the so-called critical swimming speed and the O_2_ consumption at this speed is thus the maximal sustainable aerobic metabolic rate. However, while this parameter provides a useful measure of an animal's performance, it does not qualify for a measure of MMR, which represents the maximal capacity of the O_2_ transport and utilization system and is associated with a state that is clearly not sustainable.

Zebrafish (*Danio rerio*) is a powerful model organism for studying many aspects of physiology. Their small size, high fecundity, and genetic similarity to humans make them ideal for a wide range of experimental investigations (Seth et al., 2013). Obtaining reliable values of SMR as well as MMR for zebrafish is therefore key to characterize their metabolic performance and has become increasingly relevant in physiological and biomedical studies. However, divergent values of adult zebrafish metabolic rates are found in the literature. Studies have reported highly different SMR values (Lucas et al., 2014; Huang et al., 2020) and generally low factorial aerobic scopes (Lucas et al., 2014; Huang et al., 2020; Ripley et al., 2022; Morgan et al., 2022), suggesting that MMR might have been underestimated. Here, we describe how to accurately determine SMR and MMR of zebrafish under ramping exercise.

**Fig. 1.**
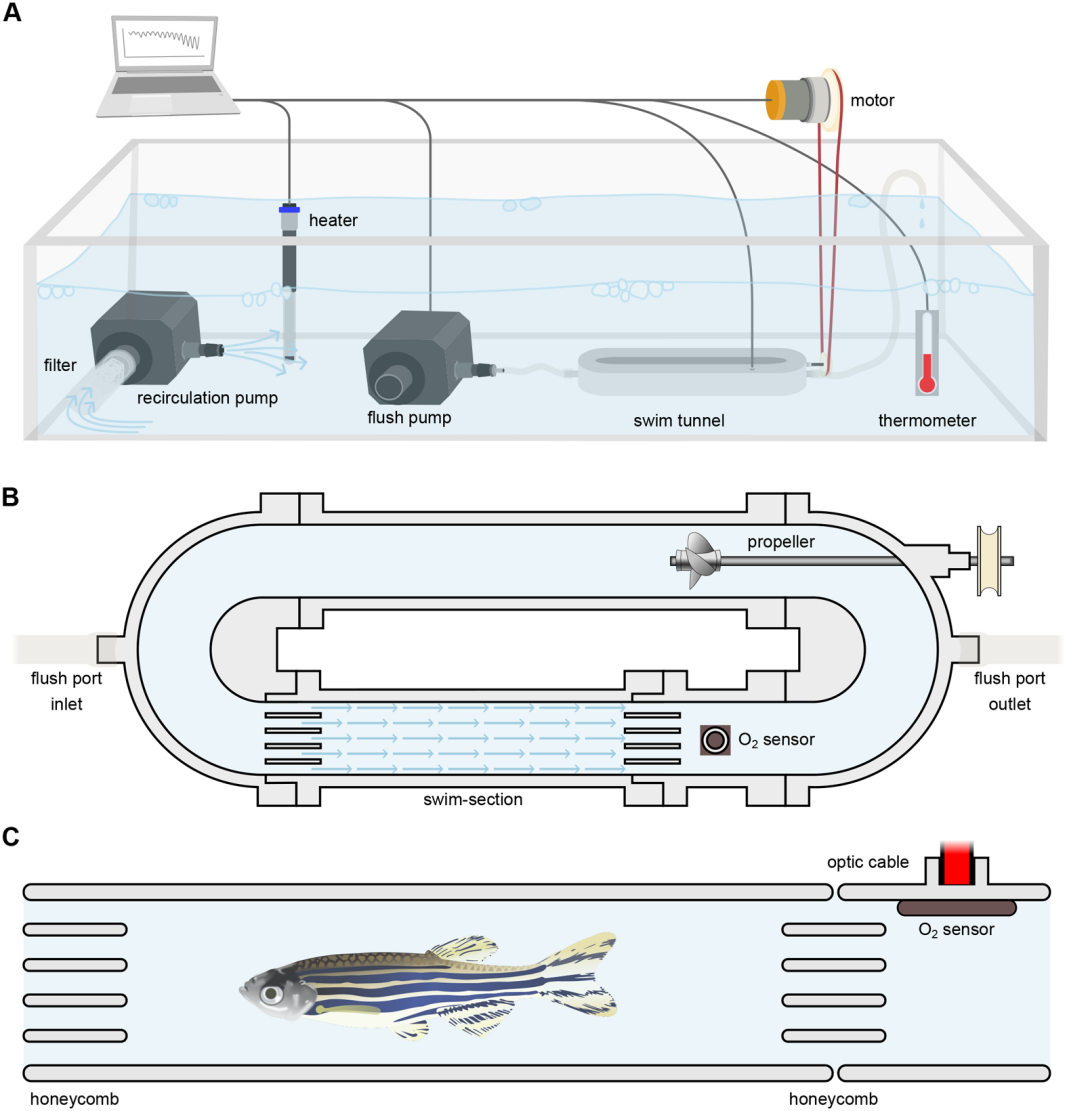
**Schematic representation of the 3D-printed intermittent-flow respirometer and swim tunnel system.** (A) The system is composed of five assembled sections and is submerged in a large aquarium equipped with a filter and recirculation pump to maintain water quality and mixing and a thermostatted heater to control water temperature. The motor driving the propeller is shown. (B) Top view of the respirometer and swim tunnel. The propeller is connected to a motor generating the water flow inside the tunnel. A honeycomb before and after the swim section ensures an even laminar flow within it, as indicated by blue arrows, and prevents the fish from escaping the swim-section. (C) Side view of the swim-section with zebrafish (not to scale). The O_2_ sensor is positioned immediately downstream of the swim-section and connected to an optic cable for data collection.

Currently there are no inexpensive, readily available solutions to measure MMR and SMR in zebrafish. In this study, we have developed a 3D-printed intermittent-flow respirometer and swim tunnel system specifically designed for accurate measure of SMR and MMR in adult zebrafish (Fig. 1). The internal volume of the swim tunnel is small enough to provide consistently accurate measurements of SMR at rest during low water flow rates, while capable of high flow rates that readily drive the fish to exhaustion, thus yielding reliable MMR measurements.

We here provide experimental protocols for measuring SMR and MMR in zebrafish in a swim tunnel and respirometer system. The 3D design is provided and can be printed and used by researchers to measure metabolic rates in zebrafish. The system can be customized further to accommodate other small fish species of various ages and sizes.

## RESULTS AND DISCUSSION

**Fig. 2.**
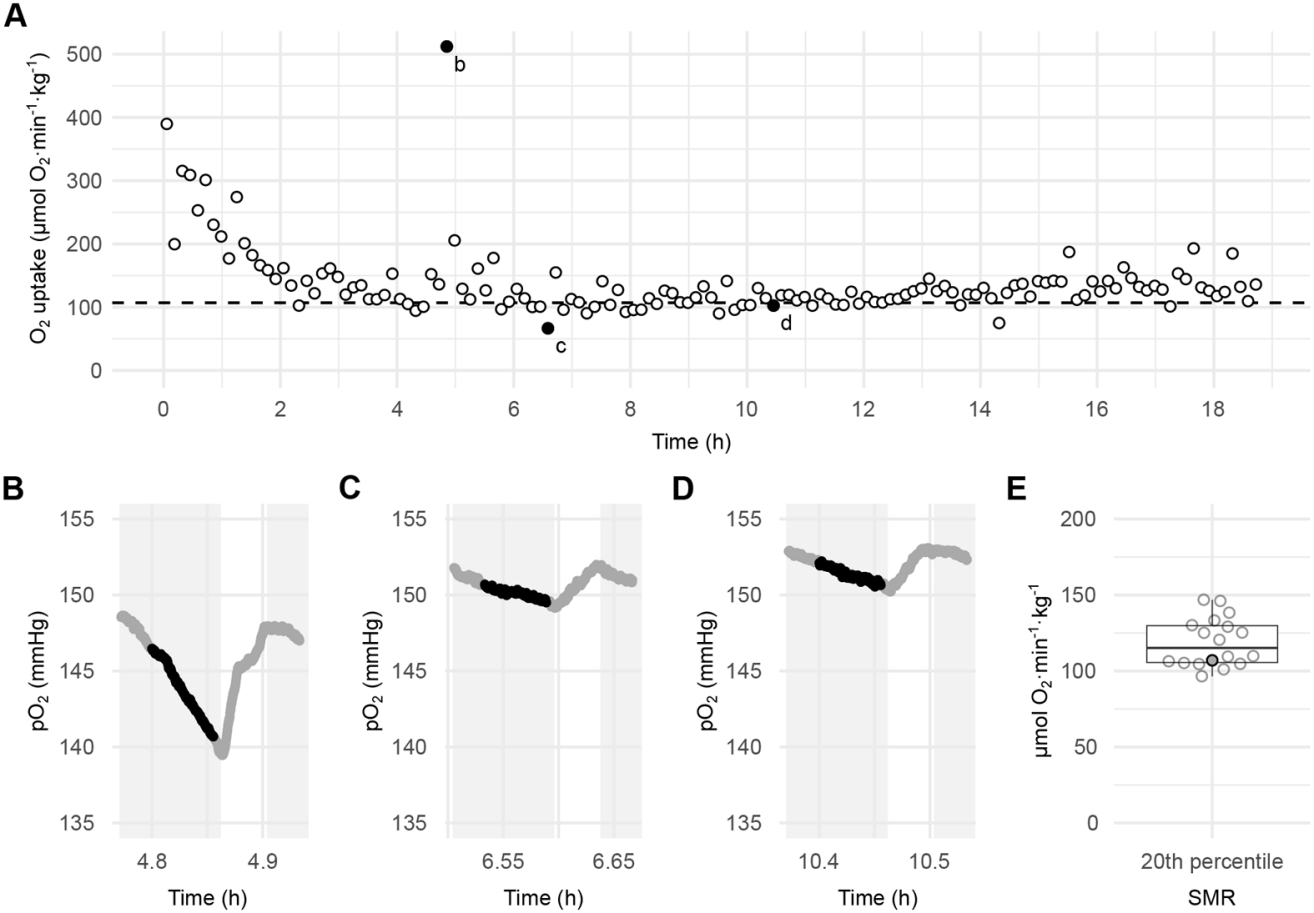
**Intermittent-flow respirometry for the determination of SMR in zebrafish.** (A) Metabolic rates of all cycles conducted over night on a representative fish. The horizontal dashed line indicates the 20th lower percentile of all values for this fish. The black dots labelled b-d indicates metabolic rates for the three examples of cycles shown in B–D, respectively. (B-D) Traces showing a high metabolic rate (B), a low metabolic rate (C), and a metabolic rate close to the SMR (D). The grey background indicates the closed periods, the white background the flushed periods, and the black dots the measured values used for determining the metabolic rates (slopes of traces). (E) Boxplot showing median, upper, and lower quartiles, and 1.5× interquartile range of SMR values from all fish (*n*=18) subjected to the protocol (*n*=18). Each dot represents an individual fish and the grey dot indicates the representative fish shown in A-D.

When measuring O_2_ consumption overnight, it took between 3 and 8 h for the metabolic rate (MR) to stabilize (Fig. 2A). Furthermore, we observed occasional spikes of high O_2_ consumption during the protocol, likely due to spontaneous activity (Fig. 2A-D). We measured a mean SMR of 119±15.9 µmol O_2_·min^−1^·kg^−1^ using intermittent-flow respirometry overnight (Fig. 2E). This value is in good agreement with the value of ∼149 µmol O_2_·min^−1^·kg^−1^ measured by Lucas et al. (2014) on similar-sized zebrafish, using a protocol with pre- and post-blank measurements, but at a slightly higher temperature of 28°C. The study by Huang et al. (2020), using a system similar to ours, reported a much higher SMR value of ∼450 µmol O_2_·min^−1^·kg^−1^ in young adult zebrafish (∼130 mg). However, they did not correct for background respiration, which may have contributed to overestimation of the SMR.

**Fig. 3.**
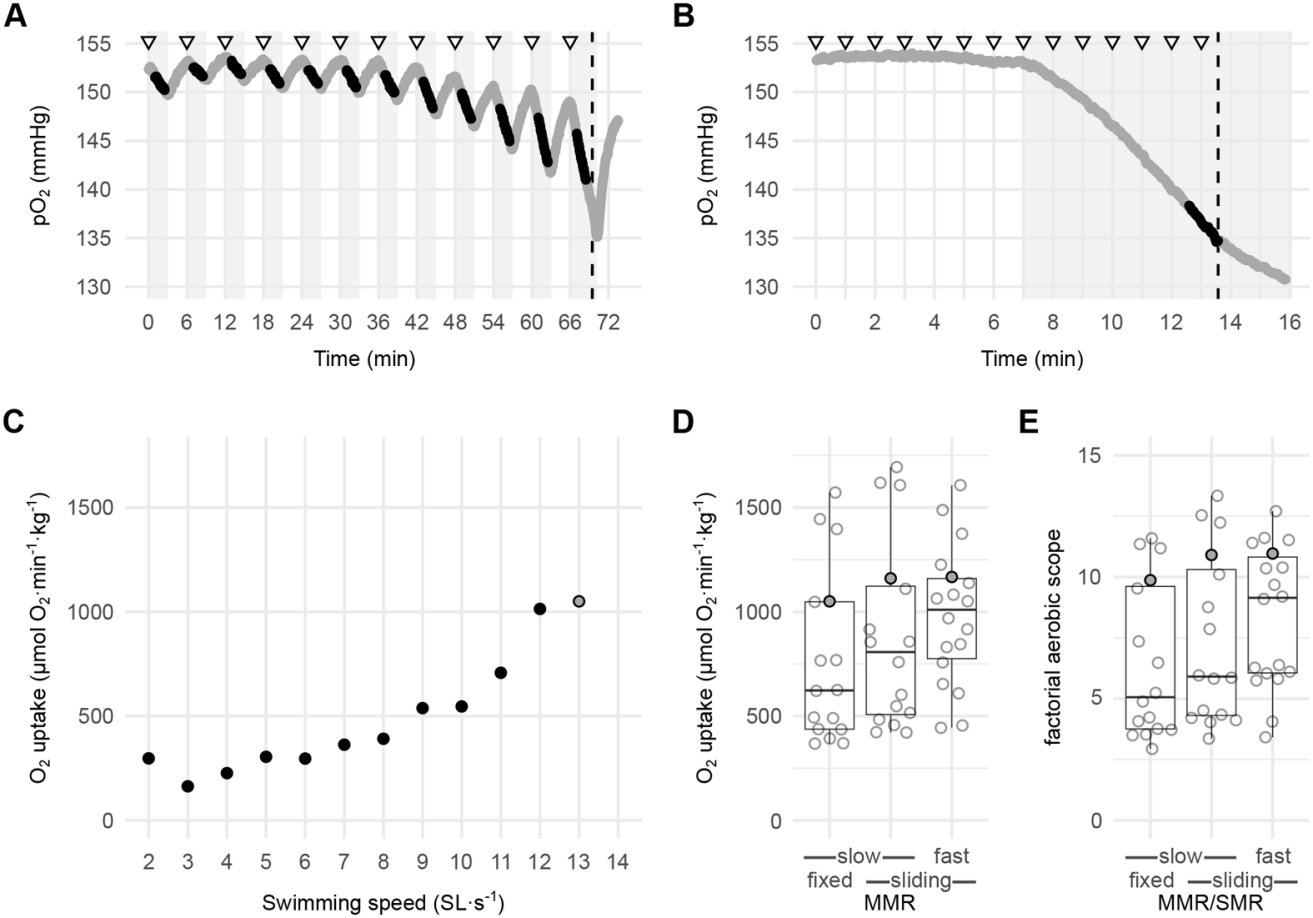
**Intermittent-flow respirometry measurement for the determination of MMR in zebrafish.** (A) Slow protocol: raw data showing pO_2_ over time for a representative fish. Triangles indicate velocity changes starting at 2 SL·s^−1^ and increased by 1 SL·s^−1^ for every 6 min. The dashed vertical line indicates when the fish yielded. The grey background indicates the closed periods, the white background the flushed periods, and the black dots the measuring period used for determining the metabolic rates. (B) Fast protocol: raw data showing pO_2_ over time for the same fish as in A. Note the difference in the x-axis scale. Triangles indicate velocity changes starting at 2 SL·s^−1^ and increased by 1 SL·s^−1^ for every min. The dashed vertical line represents when the fish yielded. The white background indicates the flushed period and the grey background the closed period. Black dots indicate the measuring period used for determining the MMR using a sliding window. (C) Metabolic rate as a function of swimming speed (SL·s^−1^) using the slow protocol and the set measuring periods in A. The grey dot at the end of the experiment is the MMR. (D) A boxplot of zebrafish (*n*=18) MMR as determined using the slow protocol (*n*=16) analyzed using a fixed or a sliding window and the fast protocol (*n*=18) analyzed using a sliding window. The grey dots indicate the fish shown in A–C. (E) Boxplot of the factorial metabolic scope (MMR/SMR) using the three different ways of measuring MMR, as described in D. The grey dots indicate the fish shown in A-C. All boxplots show median, upper and lower quartiles, and 1.5× interquartile range.

The slow protocol for measuring MMR employs a 3 min flushing period followed by a 3 min closed period for each flowrate increment (Fig. 3A,C), and this is about as fast as intermittent-flow respirometry can measure O_2_ consumption in this system. The advantage of this protocol is that pO_2_ is held as high as possible to sustain aerobic metabolism and the measurement period is long enough to estimate O_2_ consumption at each swimming speed. A potential drawback of this method is that the highest O_2_ consumption rate could be missed during the extended flush period.

The fast protocol uses a single, extended close period starting before the expected yield of the fish (Fig. 3B). By eliminating the flush period in the fast protocol, it becomes possible to increase the frequency of flow rate increases to every 1 min instead of every 6 min. This makes the protocol faster and more akin to the continuous protocol used on humans to measure MMR, where workload is rapidly ramped until reaching a plateau in O_2_ consumption (Duncan et al., 1997).

Data from the slow protocol was analyzed using a 90 s fixed window (Fig. 3A,C) or a 60 s sliding window yielding MMR of 767±411 and 877±444 µmol O_2_·min^−1^·kg^−1^, respectively (Fig. 3D). Data from the fast protocol was measured with a 60 s sliding window (Fig. 3B), yielding a MMR of 982±330 µmol O_2_·min^−1^·kg^−1^ (Fig. 3D). There was a tendency for the fast protocol to result in higher MMR values (Fig. 3D), albeit this was not significant (Kruskal–Wallis test, *P*=0.141). This is similar to the findings of Duncan et al. (1997) who compared the MMR obtained using stepwise versus continuous protocols in humans.

Our results indicate that the post-exhaustion method is not suitable for measuring MMR in zebrafish, where O_2_ consumption drops immediately after exhaustion, as shown in the fast protocol experiments (Fig. 3B). In a typical post-exhaustion experiment, by the time the fish is transferred to the respirometer, it will already have recovered considerably from exhaustion. Additionally, our data shows that low pO_2_ in the chamber did not limit MMR. We found a negative correlation between pO_2_ during the measurement period and the calculated MMR in the fast protocol (Fig. S1), indicating that the decrease in pO_2_ was a result of the high O_2_ consumption of the fish and not a limiting factor.

Using the Shapiro–Wilk normality test, MMR values were normally distributed for the fast protocol (*P*=0.936), but not the slow protocol (*P*=0.0138 and *P*=0.0207 for the fixed and sliding windows, respectively), suggesting that more consistent data are obtained by using the fast protocol. Lastly, the speed of the fast protocol provides an obvious advantage in reducing the duration of the experiment.

The factorial aerobic scope (MMR·SMR^−1^) was calculated using the three MMR methods tested here: the slow method with a fixed window (6.99±3.50), the slow method with a sliding window (8.05± 3.83), and the fast protocol using a sliding window (9.30±3.25) (Fig. 3E). Again, although the factorial aerobic scope tended to be higher when using the fast protocol, values were not significantly different (Kruskal–Wallis test, *P*=0.169). The factorial scopes in our study are higher than previously reported values in zebrafish of 2.5–4 (Lucas et al., 2014; Huang et al., 2020; Ripley et al., 2022; Morgan et al., 2022), where SMR and MMR might have been overestimated or underestimated, respectively. Conversely, our factorial scope values are similar to those of ∼7.5 found on ∼50 g brown trout (Norin and Malte, 2011, 2012) and also close to the range of 10–15 reported for mammals (Hinds et al., 1993). The advantage of our method is that it allows the researcher to completely exhaust the fish within the respirometer while simultaneously measuring O_2_ consumption.

To conclude, we have developed a 3D-printed respirometer and swim tunnel system, designed for zebrafish but likely usable for other small fishes. We show that the respirometer can measure SMR and MMR reliably on the same fish in one continuous protocol with no interruption (Fig. S2). We provide the drawings for the system (Supplementary data file 1) and this will give researchers with access to a 3D-printer an inexpensive respirometry setup for accurately measuring SMR and MMR in zebrafish. Furthermore, we demonstrate that the fast protocol of measuring MMR in zebrafish is as accurate as the slower intermittent-flow respirometry alternative. Finally, our results show that zebrafish exhibit remarkably rapid post-exhaustion recovery, which may lead to incorrect estimates of their actual MMR when using post-exhaustion methods.

## MATERIAL AND METHODS

### Zebrafish husbandry and experimentation

Zebrafish were bred in-house from AB founders from the European Zebrafish Resource Center of the Karlsruhe Institute of Technology (KIT). Fish were fed three times daily and maintained on a 14-h-light:10-h-dark cycle on recirculating housing systems at 28°C in reverse osmosis water conditioned to pH 7.2 and conductivity 700 µS using sodium bicarbonate and Instant Ocean Sea Salt. Embryos were obtained by natural crosses, reared in E3 buffer [5 mM NaCl, 0.17 mM KCl, 0.33 mM CaCl2, 0.33 mM MgSO4, 10^−5^% (w/w) methylene blue, 2 mM HEPES pH 7.2] at 28°C. All experiments were carried out according to Danish legislation and approved by the Danish Animal Experiments Inspectorate (Dyreforsøgstilsynet*,* permit number 2023-15-0201-01510). We used a total of 15 male and three female adult zebrafish with a standard length (SL) (Parichy et al., 2009) of 29±1.8 mm, a weight of 423±95.7 mg, and a condition factor of 1.67±0.225 (Bolger and Connolly, 1989) (Fig. S3).

### Respirometer and swim tunnel design

The respirometer and swim tunnel system was 3D-printed in clear resin and equipped with an O_2_ sensor and a propeller driven by a motor.

The system was designed in five sections (Fig. 1B; Fig. S4): three linear sections and two curved sections. Each rounded section has a port for inlet and outlet of water through connected tubes during flushing (Fig. 1B; Fig. S4). The system was drawn in Autodesk Inventor professional 2018 and printed using a stereolithography 3D printer (formlabs, #Form 3). The linear swim-section aimed to contain the fish was printed in clear resin (formlabs, #RS-F2-GPCL-04) while the remaining pieces were printed in durable resin (formlabs, #RS-F2-DUCL-02). Honeycombs were placed before and after the swim section to promote an even laminar flow. The O_2_ sensing spot (Oxfoil, Pyroscience GmbH, Germany) was attached to the inside of the system just downstream of the swim-section (Fig. 1B,C; Fig. S4). All connections between sections were fitted with O-rings and non-toxic vacuum grease (Dow Corning, high vacuum grease) was applied to the O-rings to ensure easy assembly and disassembly, and to prevent leaks. Once assembled, the respirometer and swim tunnel system were fastened with two screws to an aluminium stand. An electric motor (either a small RS PRO 12 V dc, 431 rpm #834-7663 or a large 12 V dc, 2329 rpm #417-9655) controls the speed of the 3D-printed propeller using a large O-ring as drive belt. The motor was mounted onto a holder fixed to the aluminium stand keeping it out of the water and its speed was controlled by a digital, programmable power supply with a resolution of 0.01 V (ELC, #ALR3203). The correlation between water flow rate and motor voltage was determined by video recording using a standard cell phone camera (iPhone 13 mini) after injecting dye into the system at different motor voltages. Approximately 0.1 ml dye (green, non-toxic fruit dye) was injected with a 1 ml syringe fitted with a gauge 23 needle extended with 3–4 cm of PE-50 catheter through one of the flush ports while the other flush port was closed. The calibration procedure yielded a linear relation between motor voltage (y) and flow speed (x) of y=0.4057x+1.5113 (R^2^=0.9917) and y=0.0689x+1.2384 (R^2^=0.9971) for the small (Fig. S5A) and the large (Fig. S5B) motor, respectively. The flow profile across the respirometer was flat right after the propeller and was still close to uniform in the swim-section (Fig. S5C,D). We did not observe a preferred spot to swim in for any of the tested fish (e.g. Movie 1).

### Standard and maximal metabolic rate

To measure O_2_ consumption rates, pO_2_ was measured over time by using a FireSting optical O_2_ meter (#FSO2-C4, Pyroscience GmbH, Germany) and data collected using Pyro Oxygen Logger version 3.312 in 1 s intervals. The entire experiment for each fish consisted of a pre-blank, SMR, MMR slow, MMR fast, and post-blank measurements (Fig. S2). All experiments were performed at 26°C. Pre-experiment background respiration (pre-blank) was determined after sterilizing (by immersion in 70% EtOH for 5 min and then allowing the EtOH to evaporate) the swim tunnel and recording O_2_ consumption for 20 min at a water flow rate of 1 cm·s^−1^. The same pre-experimental background was used for all fish as it was found to be consistently very close to zero. Post-experiment background respiration (post-blank) was measured the same way after removal of the fish. All O_2_ uptake rates were corrected for background respiration using pre- and post-experiment blank rates assuming a linear increase over time. The average background respiration contributed to a mean of 23.0±13.2% of the slopes used to determine O_2_ consumption and SMR.

Fish weight was subtracted from the internal volume of the swim tunnel to correct for its replacement of water. SMR and MMR data were analyzed using the R package respR version 2.3.1 (Harianto et al., 2019). All zebrafish were fasted for 24 h before being transferred to the swim-section of the sterilized system.

To determine SMR, O_2_ consumption was measured overnight using intermittent-flow respirometry in cycles of 5.5 min closed and 2.5 min of flushing at a flow rate of 0.5 SL·s^−1^. The flow rate of 0.5 SL·s^−1^ was low enough to allow the fish to rest at the bottom. At least 16 h of measurements (>120 periods) were recorded for each fish and the O_2_ consumption rate in each close period was calculated using linear regression over 200 s, i.e. disregarding the first 90 and the last 40 s of the close period (using the respR function calc_rate.int with a wait period of 90 s and a measure period of 200 s). SMR was defined to be the lower 20th percentile of all O_2_ consumptions rates (Chabot et al., 2016).

MMR was measured after completing the overnight SMR protocol. We tested a slow (stepwise) and a fast (continuous) protocol. Both protocols started with a water flow rate of 2 SL·s^−1^, which was increased by 1 SL·s^−1^ over a 6 min (slow) or 1 min (fast) period until the fish yielded (i.e. reached exhaustion). Yield was defined as the fish resting at the honeycomb for more than 3 s 3 times within 1 min. To test if the fish had effectively reached MMR, after 3 s of resting at the honeycomb, we applied a short interruption (∼0.5 s) of the water flow, which often resulted in the fish starting to swim again. Between the MMR protocols, the fish was given at least 1 h of rest at 0.5 SL·s^−1^.

The slow protocol used intermittent-flow respirometry for 6 min (3 min closed and 3 min flushing) per water flow rate increment resulting in one O_2_ consumption rate measurement per flow rate increment. The O_2_ consumption was then calculated using linear regression on either a fixed or a sliding window on each period. The fixed approach measured for 90 s and disregarded the first 60 and the last 30 s of the closed period (using the respR function calc_rate.int with wait=60 and measure=90). The sliding approach employed a 60 s sliding window method (using the respR function auto_rate.int functions with window size of 60 s).

In the fast protocol, the system was flushed continuously until a swimming speed of 2–4 SL·s^−1^ lower than that of yield in the slow protocol was reached. Here after, we closed the system and recorded pO_2_ continuously over time while increasing speed stepwise, until the fish yielded. The O_2_ consumption for the fast protocol was determined by applying a 60 s sliding window on the entire closed period. For all methods, MMR was defined as the highest O_2_ consumption rate observed.

### Leakage test

We measured leakage of the system by measuring O_2_ consumption in the sterilized respirometer. The chamber was filled with partly deoxygenated water. No fish were present in the chamber during this measurement. A pre- and post-blank measurement was performed as described above and was subtracted from the O_2_ consumption/leakage measured. We performed the leakage test at three different flow rates 0.5 SL·s^−1^, 5 SL·s^−1^, and 10 SL·s^−1^ (corresponding to the mean SL of the fish in this study, 29 mm). Leakage was only detectable at 10 SL·s^−1^. We found a negative correlation between chamber pO_2_ and measured O_2_ leakage with an intersection at 107 mmHg (Fig. S6). Thus, the leakage of the respirometer is negligible at normoxic condition and only worth considering if working at a low pO_2_ and a high flow rate.

### Statistics and graphs

The Kruskal–Wallis tests were performed in R using stats (version 4.3.1). All graphs were produced in R using the tidyverse package (version 2.0.0) (Wickham et al., 2019).

## Supplementary Material

10.1242/biolopen.060375_sup1 Supplementary information

Dataset 1. 3D system drawings. Drawings of all five parts of the swim tunnel and respirometry setup and propeller.
