# Supplementary material for: Assessing metabolic rates in zebrafish using a 3D-printed intermittent-flow respirometer and swim tunnel system
Source: Biol Open. 2024 Jun 18;13(6):bio060375. doi: 10.1242/bio.060375 (PMC11212631; doi:10.1242/bio.060375)
Supplement: Supplementary information [file biolopen-13-060375-s1.pdf]

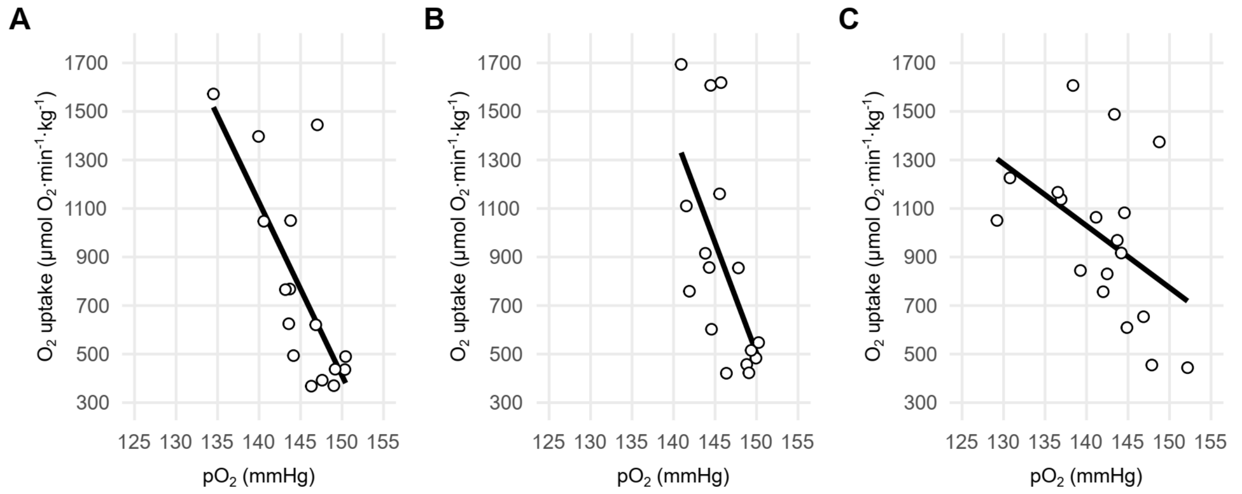

Fig. S1. Relationship between mean O_2 partial pressure (pO_2) at measurement and MMR. Dot plots depicting the relationship between pO_2 and MMR. The general trend is indicated with a linear regression line. **A)** MMR values determined using the slow protocol with fixed windows for calculating O_2 consumption (n = 16). **B)** MMR values determined using the slow protocol with a 60 s sliding window for calculating O_2 consumption (n = 16). **C)** MMR values determined using the fast protocol with a 60 s sliding window for calculating O_2 consumption (n = 18).

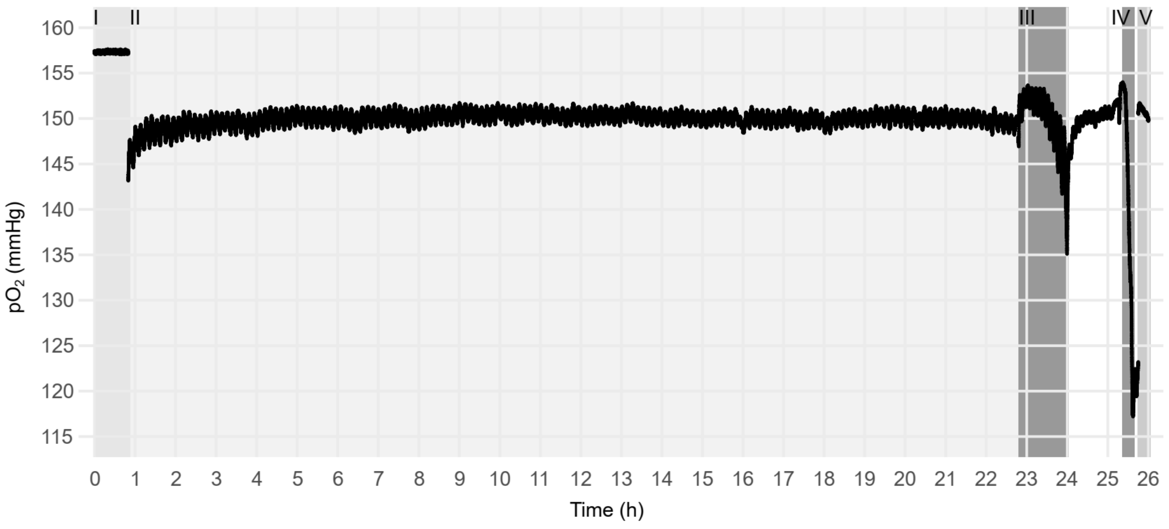

Fig. S2. Raw data showing an entire experiment of representative fish. I) Shows the pre-blank mesurement used for all fish. **II)** Shows the raw data for the SMR protocol. **III)** Shows the raw data for the slow MMR protocol. **IV)** Shows the raw data for the fast MMR protocol. **V)** Shows the raw data for the post-blank.

Biology Open • Supplementary information

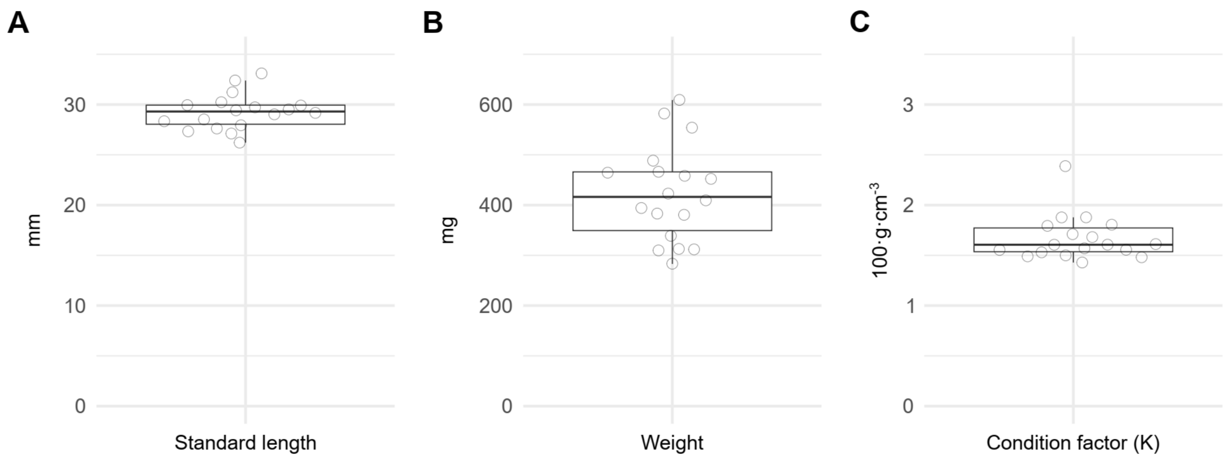

Fig. S3. Standard length, weight and condition factor of zebrafish. A) Standard length. **B)** Weight. **C)** Condition factor expressed as 100·Fultons's index, K = weight/length^3 (g·cm^{-3}). A total of 18 fish were measured. All Boxplots show median, upper, and lower quartiles, and 1.5x interquartile range.

Biology Open • Supplementary information

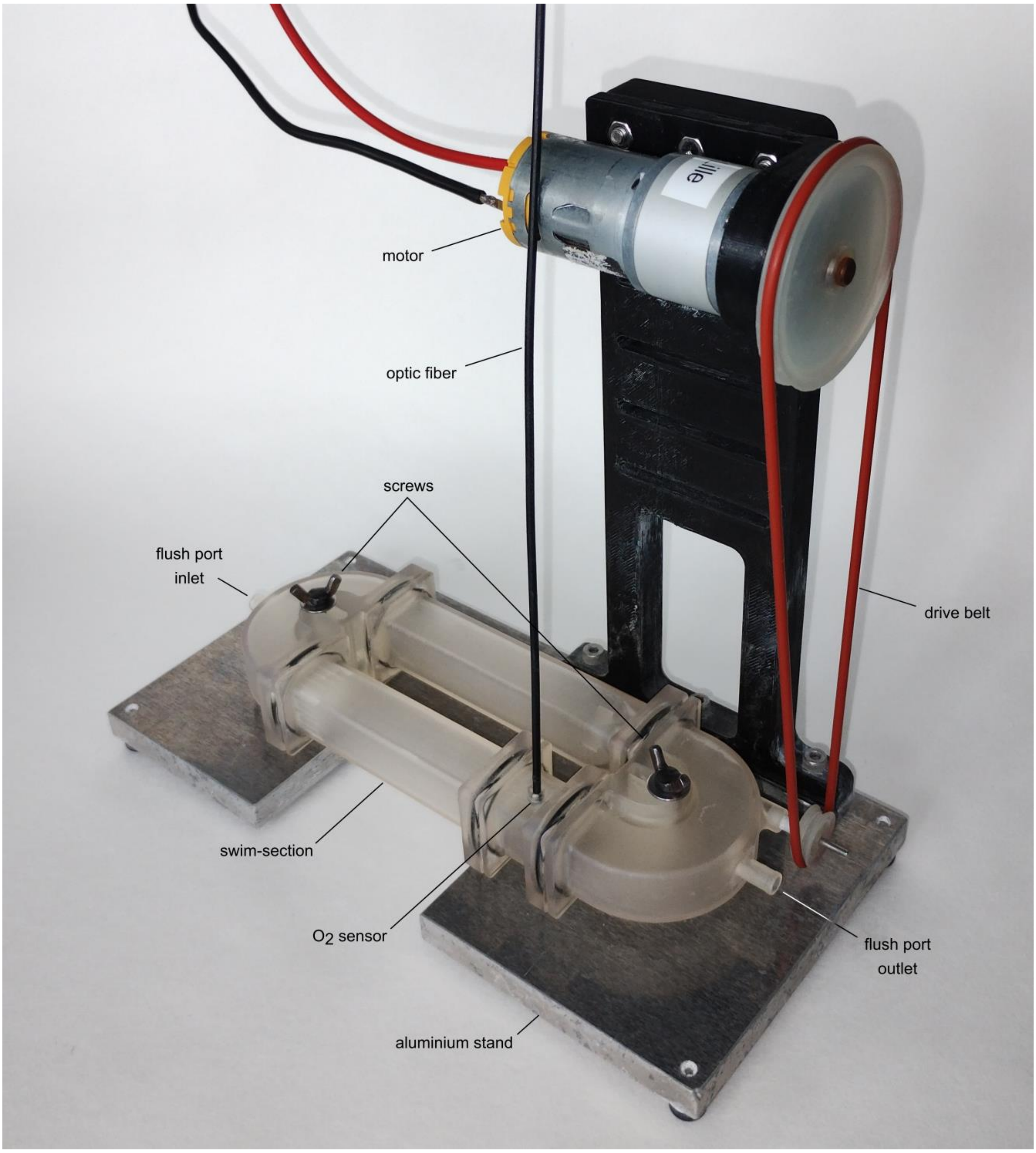

Fig. S4. Picture of the 3D-printed intermittent-flow respirometer and swim tunnel system. The system is composed of five assembled sections and fastened to and aluminium stand with two screws. The motor driving the propeller by a drive belt is attached to a holder allowing it to remain above the water. The O_2 sensor is positioned immediately downstream of the swim-section and connected to an optic cable for data collection.

Biology Open • Supplementary information

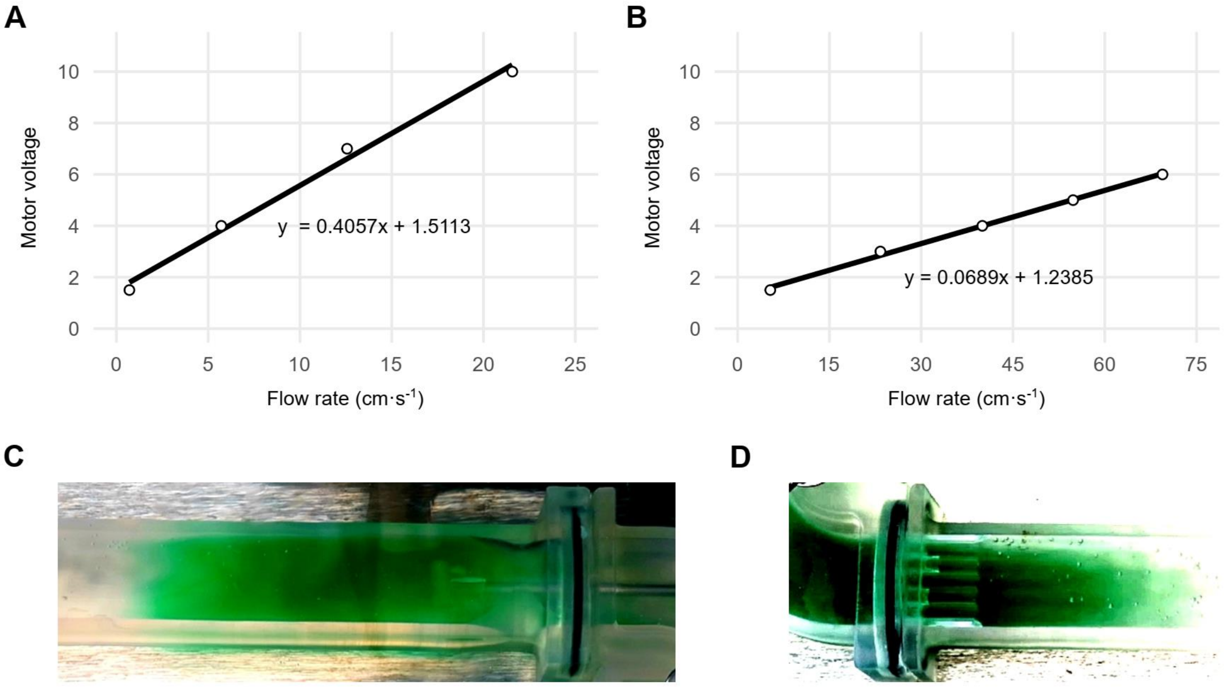

Fig. S5. Water flow rate and motor voltage in the swim tunnel. Linear relationship between motor voltage and water flow rate ($cm \cdot s^{-1}$) for the small (**A**) and big (**B**) motor setup in the swim tunnel. **C-D)** show representative images used for determining water flow rate by tracking the movement of green dye. **C)** Image showing the propellar immediately after injection of the dye. **D)** Image showing the dye passing through the first honeycomb and into the swim-section.

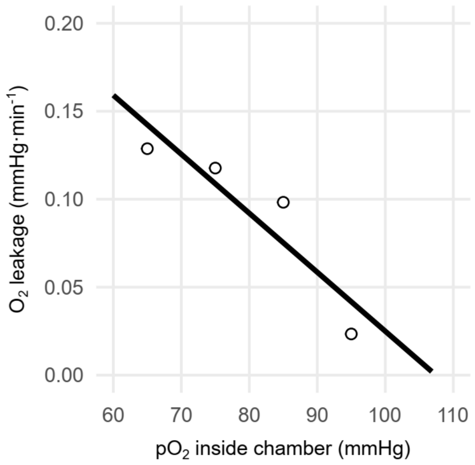

Fig. S6. O_2 Leakage in respirometer. A dot plot depicting the relationship between pO_2 inside the chamber and O_2 leakage at 10 $SL \cdot s^{-1}$. The general trend is indicated with a linear regression line.

Dataset 1. 3D system drawings. Drawings of all five parts of the swim tunnel and respirometry setup and propeller.

Available for download at
https://journals.biologists.com/bio/article-lookup/doi/10.1242/bio.060375#supplementary-data

Biology Open • Supplementary information

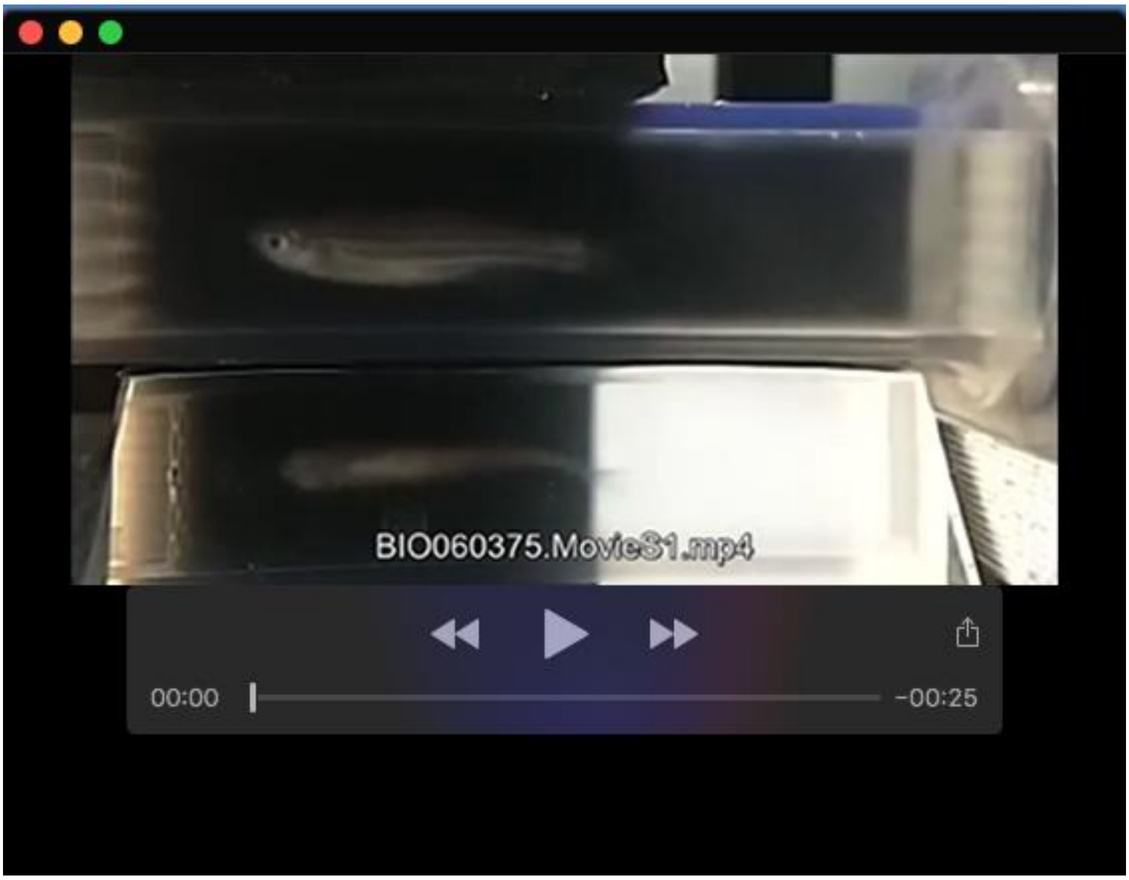

Movie 1. Recording of fish in the swim tunnel. Representative fish swimming in swim tunnel at 8 $SL{\cdot}s^{-1}$ during the fast MMR protocol.

Biology Open • Supplementary information
