# Supplementary figures and images for: Assessing metabolic rates in zebrafish using a 3D-printed intermittent-flow respirometer and swim tunnel system
Source: Biol Open. 2024 Jun 18;13(6):bio060375. doi: 10.1242/bio.060375 (PMC11212631; doi:10.1242/bio.060375)

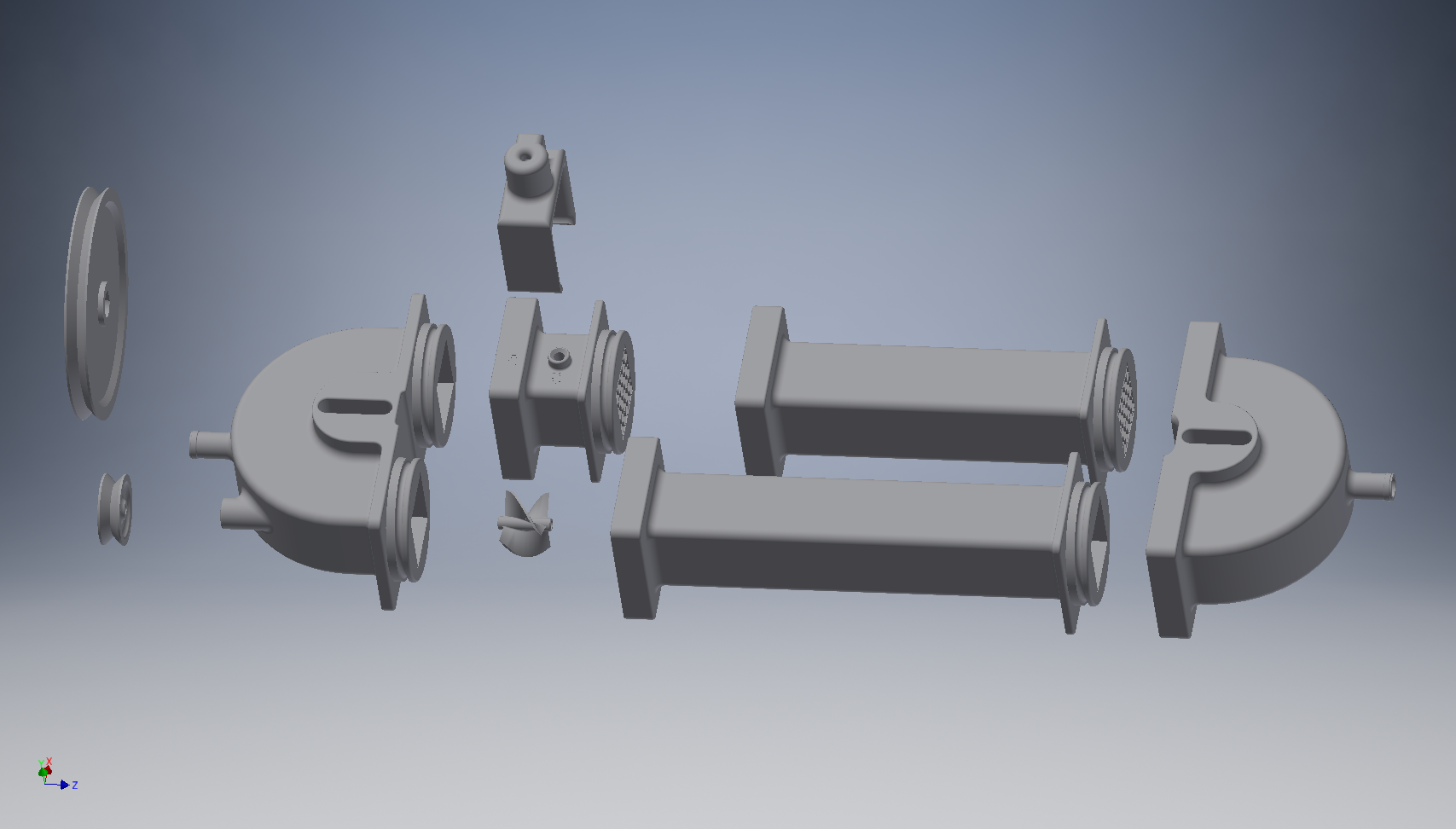

Supplement: Dataset 1. — Drawings of all five parts of the swim tunnel and respirometry setup and propeller. [file biolopen-13-060375-Daraset1.zip › 3D drawings/3D printed parts exploded view.bmp]
